# The impact of thyroid autoimmunity on pregnancy outcomes in women with unexplained infertility undergoing intrauterine insemination: a retrospective single-center cohort study and meta-analysis

**DOI:** 10.3389/fendo.2024.1359210

**Published:** 2024-03-19

**Authors:** Jiaxu Li, Jiaxin Yu, Yingqin Huang, Baoli Xie, Qianwen Hu, Nana Ma, Rongyan Qin, Jianxin Luo, Hao Wu, Ming Liao, Aiping Qin

**Affiliations:** ^1^ Reproductive Medicine Research Center, The First Affiliated Hospital of Guangxi Medical University, Nanning, Guangxi, China; ^2^ Reproductive Medicine Center, Maternity and Child Health Care of Guangxi Zhuang Autonomous Region, Nanning, Guangxi, China; ^3^ Gynecology Department, Shenzhen Luohu Hospital Group Luohu People’s Hospital, Shenzhen, Guangdong, China

**Keywords:** thyroid, autoimmunity, TSH, unexplained infertility, intrauterine insemination, pregnancy outcome

## Abstract

**Introduction:**

Infertility affects 8-12% of couples worldwide, with 15-30% classified as unexplained infertility (UI). Thyroid autoimmunity (TAI), the most common autoimmune disorder in women of reproductive age, may impact fertility and pregnancy outcomes. However, the underlying mechanism is unclear. This study focuses on intrauterine insemination (IUI) and its potential association with TAI in UI patients. It is the first meta-analysis following a comprehensive literature review to improve result accuracy and reliability.

**Methods:**

Retrospective cohort study analyzing 225 women with unexplained infertility, encompassing 542 cycles of IUI treatment. Participants were categorized into TAI+ group (N=47, N= 120 cycles) and TAI- group (N=178, N= 422 cycles). Additionally, a systematic review and meta-analyses following PRISMA guidelines were conducted, incorporating this study and two others up to June 2023, totaling 3428 IUI cycles.

**Results:**

Analysis revealed no significant difference in independent variables affecting reproductive outcomes. However, comparison based on TAI status showed significantly lower clinical pregnancy rates (OR: 0.43, P= 0.028, 95%CI: 0.20-0.93) and live birth rate (OR: 0.20, P= 0.014, 95%CI: 0.05 ~ 0.71) were significantly lower than TAI- group. There was no significant difference in pregnancy rate between the two groups (OR: 0.61, P= 0.135, 95%CI: 0.32-1.17). However, the meta-analysis combining these findings across studies did not show statistically significant differences in clinical pregnancy rates (OR:0.77, P=0.18, 95%CI: 0.53-1.13) or live birth rates (OR: 0.68, P=0.64, 95%CI: 0.13-3.47) between the TAI+ and TAI- groups.

**Discussion:**

Our retrospective cohort study found an association between TAI and reduced reproductive outcomes in women undergoing IUI for unexplained infertility. However, the meta-analysis incorporating other studies did not yield statistically significant associations. Caution is required in interpreting the relationship between thyroid autoimmunity and reproductive outcomes. Future studies should consider a broader population and a more rigorous study design to validate these findings. Clinicians dealing with women with unexplained infertility and TAI should be aware of the complexity of this field and the limitations of available evidence.

## 1 Introduction

Infertility is a global concern that challenges obstetricians and reproductive medicine specialists, and it is characterized by the inability of a couple to reach a clinical pregnancy despite engaging in regular unprotected intercourse for 12 months (1). Infertility affects 8–12% of couples of reproductive age (2), and approximately 15%-30% of infertility cases have unidentified causes, commonly referred to as idiopathic or unexplained infertility (UI) (3–5). UI does not mean the absence of a cause but rather shows the presence of one or more factors that are not yet fully understood or precisely identified. Thyroid autoimmunity (TAI), the most common autoimmune disorder in women of reproductive age, is defined as the detection of thyroid peroxidase antibodies (TPO-Ab), thyroglobulin antibodies (Tg-Ab), or both, in the serum, which may be accompanied by clinical or subclinical thyroid dysfunction. Recently, the relationship between thyroid autoantibodies and female infertility, as well as pregnancy outcomes, has become a focal point of research. However, the mechanisms by which thyroid autoantibodies affect female fertility remain unclear (6, 7). Intrauterine insemination (IUI), a commonly used assisted reproductive technique, is the preferred treatment for UI (8). Some studies have shown that the presence of thyroid autoantibodies might correlate with a heightened risk of infertility and less favorable pregnancy outcomes (9–12). The *in vitro* fertilization (IVF) or intracytoplasmic sperm injection (ICSI) procedure could potentially bypass certain theoretical sites of action in underlying autoimmune processes, leading to an underestimation of the risks tied to TAI (13, 14). We hypothesized that in women with UI, TAI may influence pregnancy outcomes following IUI. Presently, data about the influence of TAI on IUI outcomes in women with UI are notably scarce, necessitating further investigation and research. In this retrospective study, we systematically screened women who underwent IUI between January 2019, and June 2022. This study primarily investigated the influence of TAI on pregnancy, clinical pregnancy, and live birth rates. Additionally, it evaluated the effects of different thyroid-stimulating hormone (TSH) levels on reproductive outcomes. A comprehensive meta-analysis enhanced accuracy and reliability, amalgamating our results with those of other relevant published studies. This study is the first meta-analysis following a comprehensive review of the existing literature.

## 2 Materials and methods

### 2.1 Retrospective study

#### 2.1.1 Ethics statement

This study was conducted in accordance with the Declaration of Helsinki and received approval from the Ethics Committee of the First Affiliated Hospital of Guangxi Medical University (number: 2023-E554-01).

#### 2.1.2 Patients

This retrospective, single-center cohort investigation examined patients aged between 18 and 40 years who had a body mass index (BMI) between 18 and 30 years and were diagnosed with UI. From January 2019, to June 2022, these women received IUI at the Reproductive Medicine Center of the First Affiliated Hospital of Guangxi Medical University. Follow-up data will be collected until June 30, 2023. When patients opt to initiate IUI at our institution, a comprehensive thyroid function assessment is conducted. The decision to test for thyroid autoantibodies was based on an integrated patient evaluation. Once an intrauterine pregnancy is confirmed, patients who do not undergo thyroid autoantibody testing before conception usually do so during the first trimester. Given the evidence of dynamic changes in TPO-Ab and TSH levels during pregnancy, we included women whose blood samples were taken no later than pregnancy week 8, as it is highly unlikely that their thyroid status would be altered at this point (15, 16). Only patients who had undergone thyroid function and antibody determination were eligible for inclusion. We prevented inter-laboratory biases by including only patients who underwent laboratory screening at our institution. On the basis of their antibody status, patients were later categorized into a thyroid autoimmunity-positive group (TAI+ group) or a thyroid autoimmunity-negative group (TAI- group). In total, 456 infertile women were screened. The reproductive center’s electronic medical records system documented comprehensive patient histories, examination results, and pregnancy outcomes. The collected data included comprehensive details such as participants’ age, history of prior pregnancies and their outcomes, existing medical conditions, BMI, and thyroid function parameters, which encompassed thyroid-stimulating hormone (TSH), free thyroxine (fT4), and free triiodothyronine (fT3).

Additionally, thyroid autoantibody levels were assessed, along with baseline hormone levels. Estrogen (E2), progesterone (P), testosterone (T), follicle-stimulating hormone (FSH), luteinizing hormone (LH), and prolactin levels were measured on the third day of menstruation. The ovarian reserve was evaluated using anti-Müllerian hormone (AMH) levels. Carbohydrate antigen (CA125) levels were recorded to diagnose endometriosis (17). The data also captured details about the stimulation protocols used in the treatments, including the days and total amount of gonadotropin used and the resulting pregnancy outcomes. A total of 456 women were treated with IUI and completed thyroid function and antibody tests. Exclusions were made for patients with evident thyroid function abnormalities or those on thyroid-related medications (16 patients), post-thyroid surgical treatments (11 patients), uterine morphological abnormalities (24 patients, including uterine malformations, adenomyosis, and uterine fibroids), unilateral fallopian tube absence and pelvic adhesions (8 patients), ovulatory disorders and polycystic ovary syndrome (46 patients), male factors and substantial semen anomalies (98 patients), systemic autoimmune diseases (12 patients), concurrent severe systemic diseases and malignancies (6 patients), and endometriosis (10 patients). Ultimately, the study cohort comprised 225 women diagnosed with UI. No patients were lost to follow-up. All included women had regular menstrual cycles. Hysterosalpingography and ultrasonography revealed bilateral patent fallopian tubes. Ultrasonographic evaluation did not identify any considerable morphological abnormalities in the uterus or ovaries, and ovarian monitoring indicated normal ovulation. Their male partners underwent semen analyses, and mild semen abnormalities were not grounds for exclusion. Women who received donor sperm were excluded. A diagnosis of UI was established when no identifiable cause of infertility was identified despite the aforementioned evaluations.

#### 2.1.3 Laboratory procedure

All tests were conducted in our institution’s laboratory. Serum levels of estrogen, progesterone, testosterone, FSH, LH, prolactin, and β-human chorionic gonadotropin (β-hCG) were determined using the electrochemiluminescence immunoassay method on the Cobas 8000 e801 (Roche, Germany). AMH was analyzed using an electrochemiluminescence immunoassay on a Cobas 6000 e601 instrument (Roche, Germany). CA125 levels were measured using an ARCHITECT c16000 (Abbott, USA) fully automated biochemical analysis system and the Chemiflex method. Serum levels of fT3, fT4, Tg-Ab, TPO-Ab, and TSH were determined using the Beckman DXI800 (Beckman Coulter, USA) chemiluminescence immunoassay analyzer. The standard reference ranges for TSH, TPO-Ab, and Tg-Ab are 0.34-5.65 mIU/l, <30 IU/ml, and <30%, respectively. Elevated levels of TPO-Ab, Tg-Ab, or both above the established thresholds categorize patients as positive for thyroid autoimmunity (TAI+).

#### 2.1.4 IUI process

Fertility specialists typically recommend either a natural cycle regimen or a mild stimulation regimen based on a comprehensive assessment of the patient’s condition. The mild stimulation protocol involved the administration of clomiphene citrate from the third day of the menstrual cycle, either as a standalone treatment or in conjunction with human menopausal gonadotropin (hMG). Routine serum hormone measurements and ultrasonographic monitoring of follicular growth were performed during the follicular phase, and the dose of hMG was adjusted on the basis of the number of follicles, growth rate, and serum hormone levels. In contrast, the natural cycle regimen does not involve ovulation-inducing medications during the follicular phase. When ultrasonography indicates an adequate number of mature follicles (with a diameter of at least 18 millimeters) or an observed spontaneous surge in LH, either h-hCG 5000-10000 IU or r-hCG 250 μg is administered as a trigger. Then, artificial insemination was performed 32-36 hours post-hCG administration by introducing either fresh partner sperm or thawed frozen sperm into the uterine cavity using a Frydman catheter (Laboratoire, France). Sperm quality was evaluated on the basis of several parameters, including semen volume, total sperm count, sperm concentration, percentage, and total number of viable sperm, along with the percentage and total number of sperm exhibiting normal morphology. A level of β-hCG >9.5 mIU/ml is defined as indicative of pregnancy. Clinical pregnancy was defined as the detection of a gestational sac with an accompanying fetal heartbeat via ultrasound for at least four weeks following IUI. In the luteal phase and upon confirmation of pregnancy, progesterone preparations are recommended for luteal support until gestation week 12. Miscarriage is characterized by a sustained decrease in serum β-hCG levels after conception, occurring before gestation week 28, or by the absence of cardiac activity in an ultrasonographically confirmed clinical pregnancy. Births that occurred after 28 weeks of gestation were categorized as live births. The primary outcome measures included pregnancy, clinical pregnancy, and live birth rates. Pregnancy and clinical pregnancy rates were calculated as the proportion of pregnancies and clinical pregnancies per total number of cycles, respectively, whereas the live birth rate was determined as the ratio of live births to the total number of pregnancies. Our study included the pregnancy outcomes of each participant during the initial 1-3 IUI treatment cycles.

#### 2.1.5 Statistical analysis

Statistical analyses were performed using the SPSS software (version 26.0; International Business Machines Corporation, USA). Kolmogorov–Smirnov and Shapiro–Wilk tests were used to evaluate the normality of the distribution of univariate variables. All the variables followed a normal distribution and are presented as means ± standard deviation. Between-group comparisons of these data were performed using an independent sample t-test. Categorical data are expressed as numbers (percentages), and between-group comparisons for binary variables were conducted using the chi-square test. The Fisher exact test was used when categorical data had small counts. A significance level of p<0.05 was considered statistically significant.

### 2.2 Systematic review and meta-analysis

#### 2.2.1 Protocol and registration

The review adhered to the Preferred Reporting Items for Systematic Reviews and Meta-Analyses (PRISMA) guidelines (18) and was registered with PROSPERO (registration number: CRD42023473138). This study investigated whether TAI influences pregnancy outcomes in women with UI undergoing IUI.

#### 2.2.2 Search strategy

Literature searches were systematically conducted across PubMed, EMBASE, and Web of Science databases, covering the period from each database’s inception to June 2023. In PubMed, a combination of Medical Subject Headings and free-text terms were used. The search terms included “thyroid autoantibodies,” “thyroid autoimmunity,” “thyroid antibodies,” “thyroid peroxidase antibodies,” “thyroglobulin antibodies” AND “assisted reproductive technology,” “intrauterine insemination,” “infertility,” “unexplained infertility,” and “idiopathic infertility.” Similar search strategies were applied to the other databases. The search strategy was tailored for applications across various databases without imposing any language restrictions. Additionally, to identify cohort studies that examined the association between TAI and IUI outcomes, reference lists of relevant reviews and studies were meticulously examined to identify additional potentially pertinent studies.

#### 2.2.3 Study selection

Cohort studies that focused on women diagnosed with UI who underwent IUI were selected. All patients in the cohort underwent thyroid-related tests, and these studies provided data on post-treatment pregnancy outcomes and specific definitions of TAI. No restrictions were imposed on language or publication dates. However, studies involving coexisting infertility factors (e.g., polycystic ovary syndrome, female congenital uterine malformations, chromosomal disorders, and infectious diseases), thrombotic tendencies, patients with autoimmune diseases, and those undergoing treatment for thyroid disease were excluded. Moreover, redundant and irrelevant studies were omitted.

Two independent researchers evaluated the quality of the selected studies, and in the event of discrepancies, a third collaborator facilitated the resolution and consensus. We used Cochrane’s Newcastle–Ottawa Scale for quality assessment in meta-analyses, which incorporates a scoring system focusing on three primary criteria: selection of study participants, comparability of study groups, and assessment of exposure. The quality assessment checklist consists of eight items, each allocated a score of 0 or 1 point, with the “comparability of cohorts” category allowing for a maximum of 2 points. Consequently, the overall quality score for each study was evaluated on a scale ranging from 0 to 9 (19).

#### 2.2.4 Data extraction

Two reviewers independently screened the titles and abstracts of the retrieved studies to identify those that met the objectives of this systematic review. Following this initial screening, the full-text versions of these studies were obtained and independently evaluated for eligibility by the same reviewers. Data were extracted concurrently from studies that met the predetermined inclusion criteria, even if the primary objective of the studies was not the same. Electronic spreadsheets were used to record the first author, study year, research design, country, thyroid antibody detection methods and threshold values, and patient counts across various groups, number of IUI cycles performed, and reproductive outcome results. The extracted data were meticulously verified to ensure precision. Discrepancies identified during this process were rectified by carefully referring to the original publications. Furthermore, in cases where specific outcomes were not explicitly reported but could be inferred from the available raw data, these outcomes were manually computed to ensure a comprehensive analysis.

This study focused on clinical pregnancy as the primary outcome, with the number of live births considered the secondary outcome. Discrepancies concerning eligibility were resolved through consultative discussions involving a third collaborator. Notably, all the literature reviewed was in a language accessible to the research team, thereby obviating the need for translation.

#### 2.2.5 Statistical methods

The extracted data were used for meta-analysis. Regardless of the methodology employed in the original paper, Review Manager 5.3 (Cochrane Collaboration, UK) was used to analyze the data. Pregnancy outcomes were assessed using the Mantel–Haenszel odds ratio (OR) and 95% confidence interval (95%CI). Heterogeneity across studies was quantitatively measured using the I² statistic (20).

An I2 value of 0% indicates the absence of heterogeneity, whereas values of 25%, 50%, and 75% represent low, moderate, and high levels of heterogeneity, respectively. In cases where no significant heterogeneity was observed (I2<50%, p>0.05), a fixed-effect model was employed to calculate the combined effect size; otherwise, a random-effect model was used to estimate the combined effect size (21, 22).

#### 2.2.6 Biased risk

The funnel plots the distribution of effect sizes (OR) by plotting the effect sizes against standard errors (reflecting accuracy) on a logarithmic scale. We refrained from using the Egger test and instead relied on a visual inspection of the funnel plot because of the limited number of studies available for analysis (21). This decision was based on the paucity of literature within the research domain. We can assess the representation of the included studies by visually examining this chart and determining whether they cover the entire population researching the topic. Suppose asymmetry in the funnel plot is observed; in that case, it may show the existence of small study effects or other forms of bias, including, but not limited to, publication bias or biases attributable to the low quality of the studies.

## 3 Results

### 3.1 Retrospective study

#### 3.1.1 Patient’s characteristics

**Figure 1:**
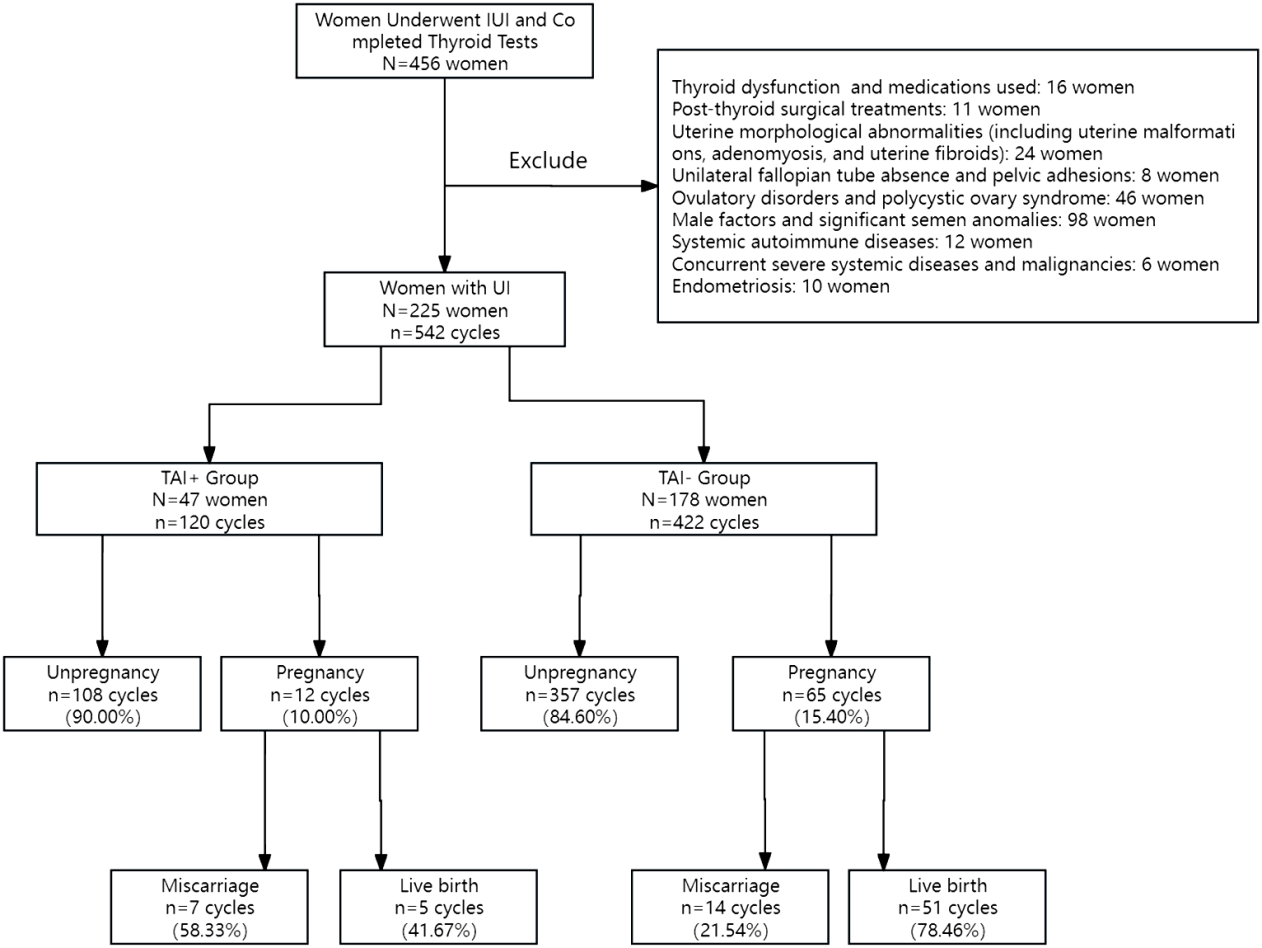
UI women selection process and pregnancy outcomes after IUI treatment.

After screening, 225 women with UI who satisfied the inclusion and exclusion criteria were enrolled in this study (**Figure 1**). These women received 542 cycles of IUI treatment in our hospital and were categorized into TAI+ group (N=47 women, n=120 cycles) and TAI- group (N=178 women, n=422 cycles) according to thyroid antibody results. In the TAI+ group, there were 14 women with both TPO-Ab and Tg-Ab positivity, 26 women with only TPO-Ab positivity, and 7 women with only Tg-Ab positivity. Each group’s baseline parameter levels (**Table 1**) and thyroid antibody levels (**Table 2**) were recorded. In addition, to further explore the impact of different TSH levels on pregnancy, we further categorized TSH levels into two groups [TSH ≥2.5 mlU/I versus (vs.) TSH <2.5 mlU/I] based on the state of TAI and compared the differences between different TSH levels. Finally, we categorized the IUI cycles into two cohorts based on TSH threshold levels to facilitate comparative analysis and evaluation of outcomes between these groups. No statistically significant variations were detected among the factors that could potentially affect fertility results.

**Table 1:** Baseline Characteristics of the 225 UI patients undergoing IUI treatment.

Patients Characteristics	TAI+ group N=47 women No.(%)	TAI- group N=178 women No.(%)	P-value*
Age(year)	32.21±4.13	31.40±3.71	0.193
BMI(kg/m²)	21.69±2.68	21.30±2.65	0.371
Infertility Years(year)	4.43±2.25	3.72±2.30	0.060
Infertility Type			
Primary Infertility	28(59.57)	119(66.85)	
Secondary Infertility	19(40.43)	59(33.15)	0.351
Basal Hormone Level			
FSH(mIU/ml)	6.52±1.78	6.3±1.68	0.438
LH(mIU/ml)	5.28±2.06	4.67±2.39	0.115
E2(pg/ml)	42.82±14.95	47.67±16.87	0.075
PROG(ng/ml)	0.30±0.17	0.33±0.29	0.523
T(ng/ml)	0.29±0.17	0.33±0.24	0.298
AMH(ng/ml)	3.17±1.79	3.58±1.99	0.201
PRL(ng/ml)	22.98±13.7	20.88±9.7	0.230
Thyroid Function			
fT3(pmol/L)	4.63±0.52	4.66±0.57	0.785
fT4(pmol/L)	10.83±1.66	10.92±1.41	0.693
TSH(mIU/L)	1.86±1.08	2.1±1.12	0.180
CA125(ng/ml)	24.31±16.23	22.49±15.88	0.486
Semen Quality			
Semen Volume(ml)	3.69±1.49	3.66±1.37	0.903
Total Number(10^6)	301.35±214.59	275.87±210.79	0.464
Concentration(10^6/ml)	88.32±63.30	78.27±53.62	0.273
Survival Count(10^6)	126.73±100.61	119.68±95.56	0.657
Survival Rate(%)	42.22±15.69	43.10±15.47	0.720
Progressive Count(10^6)	109.81±89.31	103.96±85.01	0.679
Progressive Motility(PR,%)	36.87±14.91	37.45±15.10	0.813
Normal Forms Count(10^6)	10.28±9.57	10.56±11.86	0.884
Normal Forms Rate(%)	3.20±1.55	3.70±2.11	0.132

The P-values were obtained separately through t-tests (for continuous data) and chi-square tests (for count data). Statistical significance was defined as P < 0.05.

TAI, Thyroid Autoimmunity; FSH, Follicle-Stimulating Hormone; LH, Luteinizing Hormone; E2, Estradiol; PROG, Progesterone; T, Testosterone; AMH, Anti-Mullerian Hormone; PRL, Prolactin; fT3, Free Triiodothyronine; fT4, Free Thyroxine; TSH, Thyroid-Stimulating Hormone; CA125, Cancer Antigen 125.

**Table 2:** Comparative analysis of thyroid peroxidase and thyroglobulin antibody levels among the cohorts.

TAI+: Thyroid autoimmunity positive, TAI-: Thyroid autoimmunity negative Antibodies level	TPO-Ab and TG-Ab positive group N=14 patients	TG-Ab positive group N=7 patients	TPO-Ab positive group N=26 patients	control group N=178 patients
TPO-Ab (IU/ml)	605.56±297.40	12.28±9.01	325.78±248.52	3.83±4.57
TPO-Ab median(range)	572.1 (61.51-987.32)	11.04 (1.59-27.20)	307.8 (30.39-858.00)	2.19 (0.38-24.00)
TG-Ab (%)	42.11±10.24	56.95±14.82	12.15±6.84	8.16±5.04
TG-Ab median(range)	39.56 (30.42-70.64)	59.33 (37.07-77.96)	10.32 (3.65-29.89)	6.9 (1.05-28.18)

TPO-Ab, Thyroid Peroxidase Antibodies; TG-Ab, Thyroglobulin Antibodies.

#### 3.1.2 Pregnancy outcome

##### 3.1.2.1 Primary outcome

The comparison of pregnancy outcomes based on TAI status revealed that the TAI+ group exhibited a significantly lower clinical pregnancy rate (6.67% vs. 14.22%, OR: 0.43, P=0.028, 95% CI: 0.20-0.93) and live birth rate (41.67% vs. 78.46%, OR: 0.20, P=0.014, 95% CI: 0.05-0.71) than the TAI- group. Although the pregnancy rates in the TAI+ group were observed to be lower than those in the TAI- group (10.00% vs. 15.40%), the difference between these two groups did not reach statistical significance (OR: 0.61, P=0.135, 95% CI: 0.32-1.17) (**Table 3**).

**Table 3:** Stimulation protocol and IUI outcome according to TAI status.

	TAI+ group n=120 cycles No (%)	TAI- group n=422 cycles No (%)	OR (95%CI)	P-value
Stimulation Protocol				
Natural Cycle	43 (35.83)	118 (27.96)	1.44 (0.94-2.21)	0.096
Stimulated Cycle	77 (64.17)	304 (72.04)		
Gonadotrophin Used				
Days	2.71±3.34	3.30±4.06		0.103
Dosage (IU)	221.81±286.89	322.46±678.48		0.114
IUI Outcome				
Pregnancy	12 (10.00)	65 (15.40)	0.61 (0.32-1.17)	0.135
Unpregnancy	108 (90.00)	357 (84.60)		
Clincal Pregnancy	8 (6.67)	60 (14.22)	0.43 (0.20-0.93)	0.028
Miscarriage	7 (58.33)	14 (21.54)	5.10 (1.40-18.55)	
Live Birth	5 (41.67)	51 (78.46)	0.20 (0.05-0.71)	0.014*

TAI+, Thyroid autoimmunity positive; TAI-, Thyroid autoimmunity negative.

*: FISHER exact test.

##### 3.1.2.2 Secondary outcome

In this study, we further investigated the impact of different TSH levels (TSH≥2.5 mIU/l vs. TSH <2.5 mIU/l) on pregnancy outcomes among women with UI, categorized by the positive or negative of TAI.

The TAI+ group had 35 cycles with TSH ≥2.5 mlU/I and 85 cycles with TSH <2.5 mlU/I. In the comparison between these subgroups, no statistically significant differences were observed in terms of the pregnancy rate (14.29% vs. 8.23%, P=0.329), clinical pregnancy rate (8.57% vs. 5.88%, P=0.690), or live birth rate (40.00% vs. 42.86%, P=1.00), all with p-values >0.05 (**Table 4**).

**Table 4:** IUI outcome according to TSH level.

	TAI+ group n=120 cycles		P_1_	TAI- group n=422 cycles		P_2_	Total TSH ≥2.5 mlU/I n=170 No.(%)	Total TSH <2.5 mlU/I n=372 No.(%)	OR(95%Cl)	P_3_
	TSH ≥2.5 mlU/I n_1_=35 No.(%)	TSH <2.5 mlU/I n_2_=85 No.(%)		TSH ≥2.5 mlU/I n_1_=135 No.(%)	TSH <2.5 mlU/I n_2_=287 No.(%)					
Pregnancy	5(14.29)	7(8.23)	0.329*	24(17.78)	41(14.29)	0.354	29(17.06)	48(12.90)	0.72(0.44-1.19)	0.199
Unpregnancy	30(85.71)	78(91.76)		111(82.22)	246(85.71)		141(82.94)	324(87.10)		
Clincal Pregnancy	3(8.57)	5(5.88)	0.690*	20(14.81)	40(13.94)	0.881	23(13.53)	45(12.10)	0.88(0.51-1.51)	0.640
Miscarriage	3(60.00)	4(57.14)		7(29.17)	7(17.07)		10(34.48)	11(22.92)		
Delivery	2(40.00)	3(42.86)	1.000*	17(70.83)	34(82.93)	0.252	19(65.52)	37(77.08)	1.77(0.64-4.91)	0.270

TAI+, Thyroid autoimmunity positive; TAI-, Thyroid autoimmunity negative; TSH, Thyroid-Stimulating Hormone.

*: FISHER exact test.

P_1_: P-values for comparison of different TSH levels in a TAI+ group.

P_2_: P-values for comparison of different TSH levels in a TAI- group.

P_3_: P-values were compared for all patients according to different TSH levels.

The TAI- group had 135 cycles with TSH ≥2.5 mlU/I and 287 cycles with TSH <2.5 mlU/I. Similarly, no significant differences were found between these subgroups in pregnancy rate (17.78% vs. 14.29%, P=0.354), clinical pregnancy rate (14.81% vs. 13.94%, P=0.881), or live birth rate (70.83% vs. 82.93%, P=0.252) (**Table 4**).

Finally, we categorized all IUI cycles into two groups on the basis of TSH threshold levels. There were 170 cycles in the TSH ≥2.5 mlU/I group and 372 cycles in the TSH <2.5 mlU/I group. The subgroup analysis showed no significant differences in pregnancy rate (17.06% vs. 12.90%, P=0.199, OR:0.72, 95CI: 0.44-1.19), clinical pregnancy rate (13.53% vs. 12.10%, P=0.640, OR:0.88, 95CI: 0.51-1.51), or live birth rate (65.50% vs. 77.08%, P=0.270, OR:1.77,95CI: 0.64-4.91) (**Table 4**).

### 3.2 Systematic review and meta-analysis

#### 3.2.1 Search results

**Figure 2:**
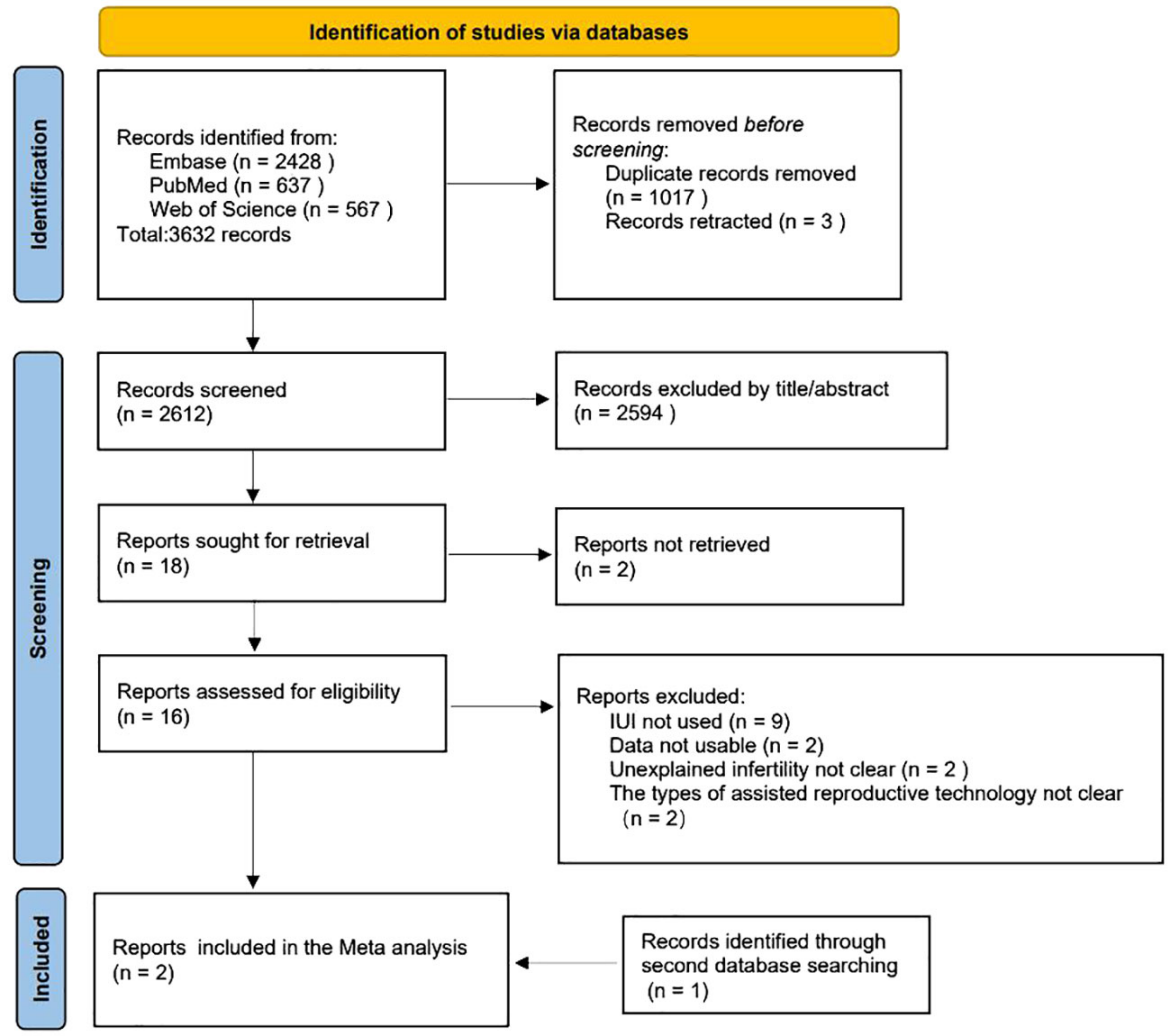
The flowchart illustrates the process of selecting studies.

A comprehensive literature search yielded 3,632 records, of which 1,017 duplicates and three retracted articles were excluded. Subsequently, the titles and abstracts of the remaining records (n=2,612) were screened on the basis of predefined criteria, including the exclusion of studies without IUI, unavailability of usable data, lack of clear definitions of UI, and ambiguity in reporting the types of assisted reproductive technology employed. This process resulted in the identification of 18 relevant reports for further study. Following the assessment for eligibility, only 2 studies (23, 24) met the inclusion criteria for subsequent meta-analysis; an additional record was identified through a secondary database search. **Figure 2** outlines the study selection process using a PRISMA flowchart (18).

#### 3.2.2 Study characteristics

**Table 5** presents the characteristics of the included studies that have been incorporated. This study and another investigation were retrospective cohort studies (24), whereas the remaining study was a double-blinded randomized prospective trial (23). In the study conducted by Turi et al., a placebo was administered to the TAI+ group, while the TAI- group served as the control. We maintain that the presence of a placebo does not modify the influence of TAI+ on pregnancy results and that the placebo and control groups are comparable, thereby meeting our criteria for literature inclusion (23).

**Table 5:** Characteristics of the included studies investigating the correlation between TAl and IUI outcome.

Study	Year	Country	Study population	Study design	Inclusion criteria	Exclusion criteria	Thyroid autoantibodies tested	Assays used and cut-off values for thyroid autoantibod	Number of women with TAI+	Number of women with TAI-	IUI protocol details	pregnancy outcome	Quality of evidence
Unuane et al.	2017	Belgium	first IUI cycle between 1 January 2010 and 31 December 2014 with follow-up of outcome until 31 December 2015	a retrospective cohort study	first IUI cycle in the center; patients who performed their laboratory screening in the institution; patients using donor sperm were not excluded.	Patients with thyroid dysfunction (TSH <0.01 mIU/l; TSH >5 mIU/l);Patients under treatment with levothyroxine or anti-thyroid drugs	TPO-Ab	Elecsys electrochemiluminescence immunoassays, reference values are<34 kIU/l for TPO-Ab	187	2956	MOH: clomiphene citrate/menopausal gonadotrophins, luteal support	CPR;MR; LBR	7
Turi et al.	2010	Turi	between January 2006 and August 2008 undergoing IUI treatment	A double-blind, randomized, prospective cohort study	A documented history of infertility for 1 year; age between 20 and 38 years; no more than 2 previous assisted reproduction treatment cycles; regular spontaneous menstrual cycles ranging from 24 to 35 days; body mass index (BMI) between 17 and 29 kg/m^2; no treatment with clomiphene citrate or gonadotropins within 1 month prior to recruitment; normal uterine cavity; and bilateral tubal patency as confirmed by hysterosalpingography and/or laparoscopy with chromosalpingography and hysteroscopy.	Clinically significant systemic disease. Ovarian or uterine disease (identified by ultrasonography). Current alcohol or drug abuse. Chronic diseases including cardiovascular, hepatic, renal, or pulmonary conditions. Antiphospholipid syndrome or other autoimmune conditions. Hyperandrogenemia or polycystic ovary syndrome. Hormone values outside the early follicular phase reference range at screening. Past severe ovarian hyperstimulation syndrome. Significant male oligospermia (below 7 million/mL with 30% motility). Severe pelvic adhesive disease (endometriosis stage III or IV). Tubal occlusion. Contraindications to gonadotropin, progesterone, or gonadotropin-releasing hormone antagonist use. Use of other investigational drugs within 1 month prior to recruitment.	TPO-Ab	electrochemiluminescence immunoassay,TPO-Ab titers were considered positive at >100 U/mL	48	50	Ovarian stimulation:intramuscular recombinant follicle-stimulating hormone,luteal support	CPR;MR	7
Present study	2023	China	January 2019 to June 2022. Follow-up data were collected up to June 30, 2023	A Retrospective Single-Center Cohort Study	Women aged 18 to 40 years with a Body Mass Index (BMI) between 18 and 30; Diagnosed with unexplained infertility; Received IUI treatment; Underwent comprehensive thyroid function and thyroid antibody assessment.	Thyroid Function Abnormalities; Post-thyroid Surgical Treatments; Uterine Morphological Abnormalities; Patients with unilateral fallopian tube absence and pelvic adhesions; ovulatory disorders and polycystic ovary syndrome; male factor infertility and significant semen anomalies; Autoimmune and Systemic Diseases; Endometriosis; Donor Sperm	TPO-Ab, Tg-Ab	chemiluminescence immunoassay analyzer, Normal values are defined as <30 IU/ml for TPO-Ab and <30% for Tg-Ab	47	178	clomiphene citrate and/or menopausal gonadotrophins, luteal support	PR,CPR,LBR	9

#### 3.2.3 Quality assessment

On the basis of the evaluation criteria of the Cochrane Newcastle–Ottawa Scale, we included the study by Turi et al. (23) in our analysis. However, during meta-analysis, we identified a potential confounding risk associated with inherent factors of the placebo; therefore, we deducted 1 point from the “Comparability” scoring item. Furthermore, as the study did not explicitly provide final follow-up dates for patients, which may influence long-term result assessment, we also deducted 1 point from the “Follow-up” scoring item. Considering all these factors collectively, this study obtained a seven-point score. Regarding the Unuane et al. (24). The inclusion of male infertility, single parents, and lesbians as indications for IUI without excluding the use of donor sperm may introduce additional confounding factors that could affect the comparability of the findings. Consequently, we deducted 1 point from the “Comparability” and “Representativeness of the Exposed Cohort” score items. The overall score for this study remains at 7 points (**Table 6**).

**Table 6:** Results of quality assessment using the Newcastle-Ottawa Scale for cohort studies.

Study	Selection				Comparability	Outcome			Scores
	Representativeness of the Exposed Cohort	Selection of the Non-Exposed Cohort	Ascertainment of Exposure	Demonstration That Outcome of Interest Was Not Present at Start of Study	Comparability of Cohorts on the Basis of the Design or Analysis	Assessment of Outcome	Was Follow-Up Long Enough for Outcomes to Occur	Adequacy of Follow Up of Cohorts	
Turi et al, 2010 (23)	★	★	★	★	★	★		★	7
Unuane et al, 2017 (24)		★	★	★	★	★	★	★	7
Present study	★	★	★	★	★★	★	★	★	9

TAI+, Thyroid autoimmunity positive; TAI-, Thyroid autoimmunity negative; IUI, Intrauterine Insemination.

#### 3.2.4 Results of syntheses

##### 3.2.4.1 Clinical pregnancy rate

**Figure 3:**
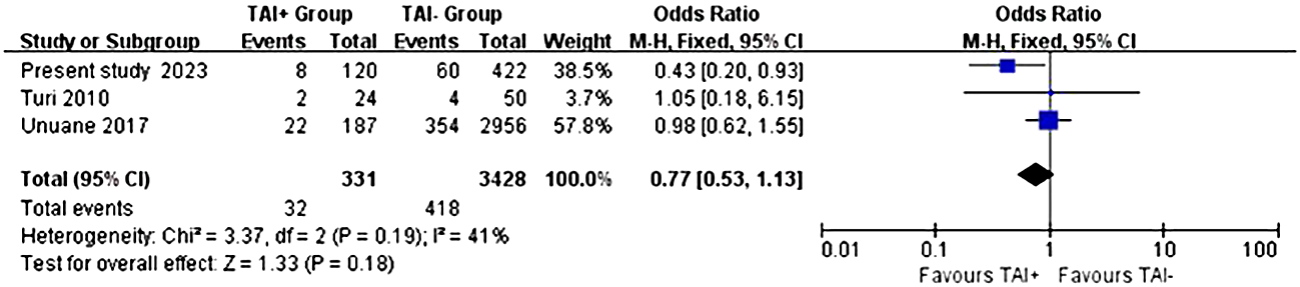
Fixed-effect meta-analysis investigating the impact of TAI status on the clinical pregnancy.

**Figure 4:**
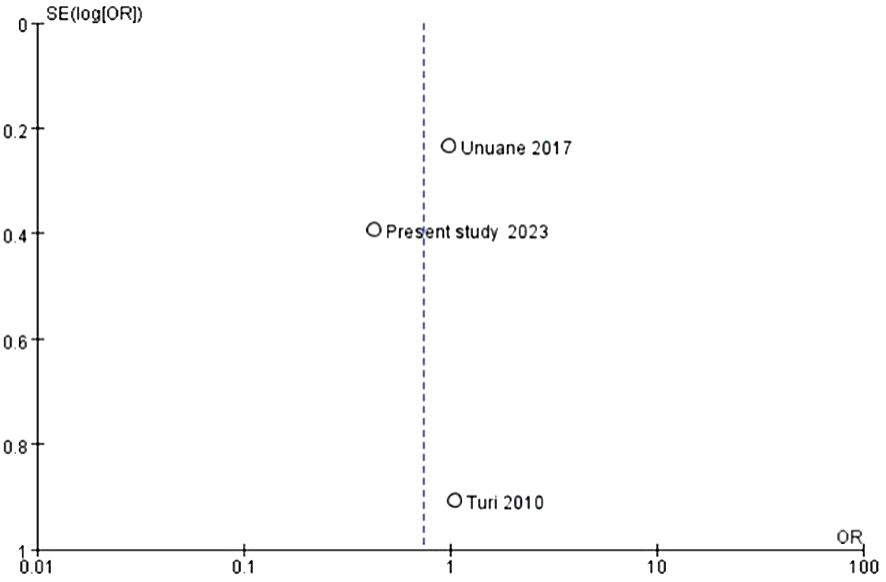
Evaluation of publication bias in studies of the effect of thyroid autoimmune status on clinical pregnancy.

This analysis incorporated 3759 female participants by considering three studies (23, 24) that included this study. Our results demonstrate that the clinical pregnancy rates do not exhibit a statistically significant disparity between the TAI+ and TAI- groups (OR:0.77, P=0.18, 95% CI: 0.53-1.13) (**Figure 3**). A fixed-effects model was used to calculate the pooled effect estimate, as indicated by the relatively low I2 value of 41%, demonstrating minimal heterogeneity (**Figure 3**). These results show that TAI status may not be pivotal in determining clinical pregnancy outcomes. The funnel plot showed no evidence of asymmetry across studies (**Figure 4**).

##### 3.2.4.2 Live birth rate

**Figure 5:**
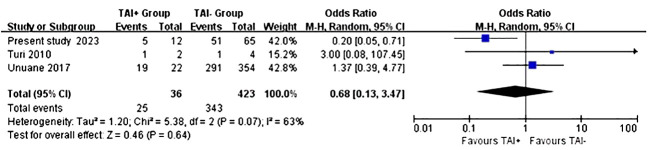
Random-effects meta-analysis investigating the impact of TAI status on the live birth. .

**Figure 6:**
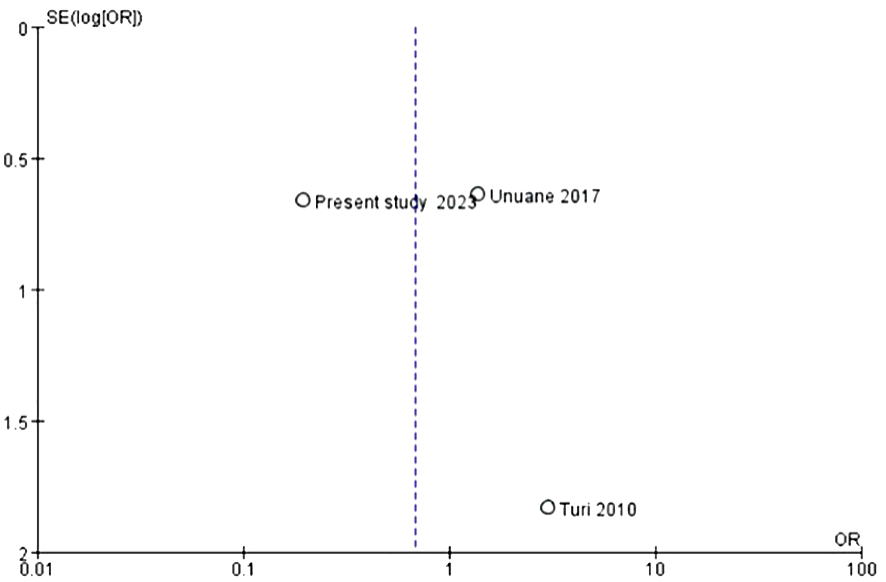
Evaluation of publication bias in studies of the effect of thyroid autoimmune status on live birth.

In terms of live birth rates, the same three studies involving a total of 459 women were included. The analysis findings indicated that there were no noteworthy disparities in the odds ratios and confidence intervals concerning the live birth rates when comparing the TAI+ group with the TAI- group (OR: 0.68, P=0.64, 95% CI: 0.13-3.47) (**Figure 5**). Considering the moderate heterogeneity indicated by the I2 value of 63%, a random-effects model was employed to derive the pooled effect estimate (**Figure 5**). The presence of bias in the funnel plot may have impeded our ability to conduct a comprehensive analysis, given the limited sample size available for inclusion (**Figure 6**).

## 4 Discussion

Our findings demonstrate a significant correlation between TAI positivity and reduced clinical pregnancy rates and live births. Although TAI-positive women exhibited lower pregnancy rates than their TAI-negative counterparts, this difference was insignificant. We assume that TAI may directly or indirectly affect a woman’s fertility. However, the available evidence does not support this conclusion (23, 24).

Unuane et al. reported that TAI did not affect live birth rates or clinical pregnancy rates in women with normal thyroid function who underwent IUI treatment (24). Our research contradicts this finding, which may be attributed to biased patient selection. Turi et al. reached a similar conclusion due to the small sample size, although they severely limited the inclusion criteria (23). There is no consensus within the academic community regarding the precise definition of UI. Generally, UI is regarded as a diagnosis of exclusion via assessments such as fallopian tube examination, ovulation monitoring, and semen analysis without identifying any potential cause (3, 5).

The presence of TAI is linked to a higher likelihood of experiencing various reproductive challenges, e.g., UI, miscarriage, repeated miscarriages, premature birth, and postpartum thyroiditis in mothers (10, 25, 26). A meta-analysis by Van et al. indicated an increased risk of UI in positive thyroid antibodies compared with negative thyroid antibodies (OR=1.5, P=0.02, 95%CI: 1.1-2.0) (12). In most studies, TAI is defined on the basis of the results of TPO-Ab tests. However, relying solely on TPO-Ab as a diagnostic standard for TAI may lead to erroneous overlooking approximately 5% of TAI cases (27). Therefore, we broadened the scope of TAI by incorporating Tg-Abs in addition to TPO-Abs. Numerous research findings have consistently indicated a higher occurrence of TAI among females who have been diagnosed with infertility (28).

Some studies have highlighted the broad systemic immune abnormalities in which TAI may be involved, one of the possible causes is the follicular microenvironment of women with TAI experiences a substantial immune imbalance, possibly leading to adverse follicles quality in those undergoing ART therapy (29, 30). Besides, the impact of the immune abnormalities in intrauterine environment, which may potentially affect the successful implantation of embryos, consequently influences the outcome of gestation (31, 32). The existence of antiphospholipid syndrome is associated with repeated reproductive failure, and the existing literature confirms its correlation with TAI (33). There is another explanation that cross-reactivity between thyroid antibodies and antigens in eggs, embryos, and placental tissues may contribute to implantation failure and pregnancy-related complications. This immune response can lead to misidentification and subsequent attacks on these crucial reproductive tissues, impacting the pregnancy (14, 34). Other types of antithyroid antibodies possess the ability to traverse the placental barrier and affect fetal thyroid function, thereby presenting a heightened risk to pregnancy (35). In summary, TAI may affect follicle and embryo quality and development, and may disrupt specific aspects of embryo implantation, ultimately leading to miscarriage that remains incompletely understood in terms of its exact pathological mechanisms.

There is currently insufficient evidence to recommend thyroid antibody screening (36). The latest guidelines on UI from the European Society of Reproduction and Embryology indicate that testing for thyroid antibodies and other autoimmune diseases is not necessary in cases of UI (5). While there is an ongoing debate surrounding the testing of TAI in women experiencing infertility, we emphasize the importance of considering thyroid immune status in managing infertile patients and closely monitoring and addressing these conditions throughout pregnancy.

The effect of subclinical hypothyroidism (SCH), particularly with TAI, on reproductive outcomes remains controversial. Endocrine abnormalities may arise in individuals with TAI because of elevated TSH levels, thereby contributing to infertility (7, 37). In the population with normal thyroid function, even preconception TSH values within the upper limit of normal range do not significantly affect IUI outcomes (24, 38–40). According to the 2015 guidelines issued by the ASRM, there is currently insufficient evidence to establish a direct correlation between TSH levels ranging from 2.5 to 4 mIU/L and the risk of miscarriage or pregnancy-related complications (41). Our findings indicate that irrespective of TAI status, when TSH levels fell within the normal range, specifically using a TSH threshold of 2.5 mIU/L to assess pregnancy outcomes, there was no statistically significant difference observed in terms of pregnancy rate, clinical pregnancy rate, and live birth rate between the two groups. These results remained consistent even when disregarding the presence or absence of TAI. These findings align with those of Unuane et al.’s study (24), which failed to establish a definitive link between TSH levels within the higher normal range and reproductive outcomes.

Published reviews and meta-analyses did not find an association between TAI and pregnancy outcomes (13, 42, 43). A recent systematic review and meta-analysis conducted by Busnelli et al. proposed that women with TAI face an increased risk of adverse outcomes when undergoing ART (44). A network meta-analysis by Wang et al. indicated that there is currently insufficient evidence to support significant differences in live birth rates for UI treatment options such as ovulation stimulation (OS), IUI, OS-IUI combination, and IVF/ICSI (45). There has been a lot of research on assisted reproductive technology for TAI or UI; however, the scarcity of studies exclusively involving UI women with TAI undergoing IUI limits the ability to draw clinical inferences. Recent emphasis on treatment strategies for UI with TAI highlights the need for further research to elucidate the relative effects of different interventions.

According to our survey, there is a dearth of systematic analyses on the effects of TAI in UI treatment for incognizant patients. Therefore, we conducted an initial comprehensive systematic evaluation and meta-analysis on the basis of available data to assess the impact of TAI on pregnancy outcomes in UI women treated with IUI. Our analysis, including three studies, revealed no significant difference in clinical pregnancy rates and live birth rates between the TAI-positive and TAI-negative groups. While the funnel plot did not indicate any signs of bias, we acknowledge that its presence cannot be completely excluded due to limited trial inclusion. Although our analysis does not definitively establish clinical pregnancy and childbirth rates, these findings still hold substantial importance and value within the research field.

For couples experiencing UI, the ASRM recommends initial attempts at ovarian stimulation combined with IUI treatment, typically involving three to four treatment cycles. If pregnancy is not achieved following these initial approaches, the ASRM recommends considering IVF as an alternative to expedite conception (3). This study demonstrates that IVF/ICSI may be a better treatment option for women with UI and TAI. This view is also supported by the European Thyroid Association, which recommends IVF/ICSI as a treatment strategy for infertile women with TAI (46). This recommendation is based on a comprehensive evaluation of treatment efficacy and patient impact and aims to provide a more effective therapeutic pathway for individuals with UI.

Most research has focused on infertility treatment techniques rather than investigating the underlying causes. Trial immunotherapy has garnered considerable attention in the realm of infertility treatment, particularly considering a burgeoning body of research indicating that levothyroxine monotherapy may not suffice to enhance fertility or optimize pregnancy outcomes (47–49). Turi et al. (23) used prednisone to intervene in TAI-positive women treated with IUI for UI. Their results demonstrated that the prophylactic administration of prednisone was significantly associated with higher pregnancy rates. Furthermore, combination therapy with prednisolone enhances clinical pregnancy rates and reduces miscarriage rates in women with TAI undergoing IVF (50). However, a study involving women with TAI who underwent IVF/ICSI indicated that adjunctive therapy with aspirin and prednisone after embryo transfer may not be necessary for fresh or frozen embryos (51). This finding demonstrates that targeted pharmacological interventions may prove advantageous in improving reproductive outcomes among patients with TAI. These treatment options are primarily supported by limited evidence from small-scale studies, warranting additional clinical trials and scientific validation to ensure their safety and effectiveness.

Our retrospective study strictly adhered to the diagnostic criteria for UI and thoroughly documented the inclusion criteria for patients. However, a major limitation of this study is its small sample size, which may compromise the statistical power and limit the generalizability and reliability of the findings. Furthermore, we posit that discrepancies in meta-analyses arise from study heterogeneity, limited publication, inadequate study designs, and diverse causes of infertility; all these factors have the potential to undermine the reliability of meta-analytical findings. Nevertheless, given these limitations, definitive conclusions cannot be drawn.

In summary, the presence of TAI in euthyroid women with UI may potentially result in adverse pregnancy outcomes when undergoing IUI, although statistical significance was not reached in our meta-analysis. To validate these findings, future research should prioritize conducting a greater number of multicenter prospective patient cohort studies that are meticulously screened, along with high-quality systematic reviews and comprehensive meta-analyses to thoroughly evaluate the impact of TAI on pregnancy outcomes in cases of UI. Considering IUI treatment for UI, it is advisable to screen for TAI as this not only assists in selecting the most suitable assisted reproduction method but also facilitates the implementation of appropriate treatment strategies.

## Data availability statement

The original contributions presented in the study are included in the article/supplementary material. Further inquiries can be directed to the corresponding authors.

## Ethics statement

The studies involving humans were approved by Ethics Committee of the First Affiliated Hospital of Guangxi Medical University. The studies were conducted in accordance with the local legislation and institutional requirements. The ethics committee/institutional review board waived the requirement of written informed consent for participation from the participants or the participants’ legal guardians/next of kin because this is a retrospective study, no informed consent is required.

## Author contributions

JL: Conceptualization, Data curation, Formal analysis, Methodology, Software, Supervision, Validation, Visualization, Writing – original draft, Writing – review & editing. JY: Data curation, Formal analysis, Methodology, Visualization, Writing – original draft, Writing – review & editing. YH: Data curation, Formal analysis, Project administration, Software, Writing – original draft, Writing – review & editing. BX: Data curation, Formal analysis, Project administration, Writing – review & editing. QH: Data curation, Methodology, Resources, Writing – review & editing. NM: Validation, Visualization, Writing – review & editing. RQ: Funding acquisition, Resources, Writing – review & editing. JL: Data curation, Formal analysis, Visualization, Writing – review & editing. HW: Software, Visualization, Writing – review & editing. ML: Methodology, Supervision, Writing – original draft, Writing – review & editing. AQ: Conceptualization, Data curation, Funding acquisition, Methodology, Project administration, Resources, Supervision, Validation, Writing – original draft, Writing – review & editing.
